# Magnetic Particle Imaging Distinguishes Viable and Damaged Cells by Exploiting Distinct Magnetic Signatures of Internalized Nanoparticles

**DOI:** 10.1002/smsc.202500601

**Published:** 2026-04-07

**Authors:** Lena Kampen, Olaf Kosch, Anke Stach, Nike D. C. Fiebig, Norbert Löwa, Frank Wiekhorst, Antje Ludwig, Amani Remmo

**Affiliations:** ^1^ Deutsches Herzzentrum der Charité Department of Cardiology Angiology and Intensive Care Medicine Berlin Germany; ^2^ Charité – Universitätsmedizin Berlin Corporate member of Freie Universität Berlin and Humboldt‐Universität zu Berlin Department of Cardiology Angiology and Intensive Care Medicine Berlin Germany; ^3^ DZHK (German Centre for Cardiovascular Research) Berlin Germany; ^4^ Physikalisch‐Technische Bundesanstalt Working Group 8.23 Metrology for Magnetic Nanoparticles Berlin Germany

**Keywords:** cell physiology imaging, functional cell tracking, immune cell tracking, magnetic particle imaging, magnetic particle spectroscopy

## Abstract

Magnetic particle imaging (MPI) enables quantitative and highly sensitive detection of magnetically labeled cells. Yet, MPI has mainly been applied to spatial cell tracking rather than assessing cell physiology. Here, MPI is expanded toward functional cell tracking by exploiting cell physiology‐dependent magnetic signatures using color MPI. THP1 cells were labeled with a citrate‐coated iron oxide nanoparticle system (Synomag‐COOH, SynC). Labeling preserved immune cell functions, without inducing oxidative stress, inflammasome activation, or altering adhesion to the inflamed endothelium. Magnetic particle spectroscopy (MPS) revealed that intracellular processing and extracellular oxidative stress modify the magnetic signature of internalized nanoparticles, reflected in changes to key MPS parameters: *A*_3_, *A*_5_ */A*_3_ ratio, and *ϕ*_3_. Nanoparticles internalized by dividing and nondividing cells exhibited opposite *A*_5_ */A*_3_ trends over 72 h, which indicated that cell type and intracellular processing distinctly modulate the magnetic signatures. Exposure to extracellular oxidative stress induced a distinct magnetic signature of internalized SynC. Color MPI successfully visualized these magnetic signature differences between oxidatively damaged and viable cells within mixed populations. These findings demonstrated that cell physiological modulation of the magnetic signatures of internalized nanoparticles enables simultaneous mapping of cell location and physiology. This established a proof‐of‐principle for a magnetic nanoparticle‐based approach that might enable monitoring of cell state during regenerative and immunotherapies.

## Graphical abstract

Magnetic particle imaging (MPI) is extended beyond spatial cell tracking to functional imaging by exploiting cell physiology‐dependent magnetic signatures of magnetically labeled immune cells. Cellular damage, induced by extracellular oxidative stress, altered the magnetic signature of internalized nanoparticles, enabling color MPI to distinguish viable from damaged cells. This established a foundation for noninvasive monitoring of cell physiology in cell‐based therapies.

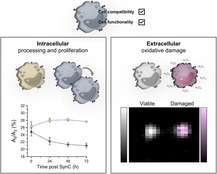

## 1 Introduction

In vivo cell tracking monitors the migration, distribution, and survival of therapeutic cells using noninvasive imaging techniques. By mapping how and where injected cells persist over time, the effectiveness of cell‐based therapies can be directly assessed. This is particularly critical for immunotherapies, where therapeutic immune cells are introduced into the circulation and must home to sites of inflammation to exert their therapeutic effects [1, 2]. Understanding the fate of therapeutic immune cells is critical for optimizing immunotherapies, especially given that inflammation is a hallmark of numerous diseases, including cancer, atherosclerosis, and autoimmune disorders [3, 4, 5]. Effective translation of cell‐based immunotherapies requires noninvasive imaging modalities that are sensitive, quantitative, and suitable for longitudinal cell tracking in vivo [6]. Current approaches include positron emission tomography (PET) [7, 8], single‐photon emission computed tomography (SPECT) [9, 10], magnetic resonance imaging (MRI) [11, 12], and magnetic particle imaging (MPI) [13, 14, 15]. Although PET and SPECT achieve high sensitivity, they rely on radioactive tracers that may compromise cell viability and limit long‐term cell tracking [1, 6]. While MRI does not use ionizing radiation, its sensitivity is limited by background signals from surrounding tissues [16, 17]. In contrast, MPI directly detects the response of magnetic nanoparticle tracers, providing a quantitative and inherently background‐free readout [15, 18]. MPI has demonstrated sensitivity for as few as 200 labeled cells in vitro, making it uniquely suited for tracking immune cells, though in vivo sensitivity remains a challenge [17, 18, 19, 20].

A key prerequisite for reliable MPI cell tracking is the efficient internalization of cell‐compatible tracers [17, 21]. Iron oxide nanoparticles are typically engulfed into the endosomal–lysosomal pathway, where the acidic environment induces progressive degradation. During this process, released iron is predominantly incorporated into physiological iron metabolism pathways, resulting in a gradual loss of magnetic anisotropy and signal over time, as shown for citrate‐coated iron oxide nanoparticles [22, 23, 24]. However, depending on the cellular context and iron handling capacity, a transient increase in free iron may also promote the formation of reactive oxygen species (ROS) [25, 26, 27, 28]. Such perturbations may alter cell functions, including altered adherence to the endothelium, which is essential for efficient homing to inflammatory sites [29, 30]. An effect of nanoparticles on cell functionality could confound MPI readouts by reflecting tracer‐induced artifacts rather than true physiological behavior. Avoiding these effects is critical for the accurate interpretation of MPI‐based cell tracking data [31, 32, 33].

While tracers might influence cellular physiology, the physiological state of the cell could, in turn, affect the magnetic properties of MPI tracers. It was previously shown that MPI tracers are not magnetically inert within biological environments [34, 35, 36, 37]. Citrate‐coated iron oxide nanoparticles exhibit distinct MPI signatures upon cell interaction, enabling the distinction between free and cell‐associated tracers [37]. We hypothesize that intracellular processes and extracellular factors also affect the magnetic signature of internalized nanoparticles. Distinguishing these specific magnetic signatures and assigning them different colors during image reconstruction could enable an approach termed color MPI [38, 39, 40]. This raises the intriguing possibility that MPI could move beyond cell localization toward cell physiological readouts. Key cell physiological processes to consider include lysosomal tracer degradation during cell tracking and extracellular oxidative stress present at inflammatory sites [41, 42].

We used Synomag‐COOH (SynC), a citrate‐coated iron oxide nanoparticle system that is rapidly internalized by immune cells and validated as a robust MPI cell tracking tracer [23, 43]. We first evaluated whether SynC labeling perturbs key monocyte functions by assessing oxidative stress, inflammasome activation, and adhesion to endothelial cells under basal and inflammatory conditions. We then examined how intracellular processing of SynC during longitudinal measurements alters its magnetic signature, detected with magnetic particle spectroscopy (MPS). To mimic the effect of the inflamed environment, we investigated the impact of high extracellular oxidative stress on the magnetic parameters of internalized SynC. Finally, we tested whether these magnetic signatures can be distinguished with color MPI, thereby establishing the basis for MPI‐based imaging of cell physiology.

## 2 Results and Discussion

### 2.1 SynC Labeling Does Not Impair Immune Cell Function

**FIGURE 1:**
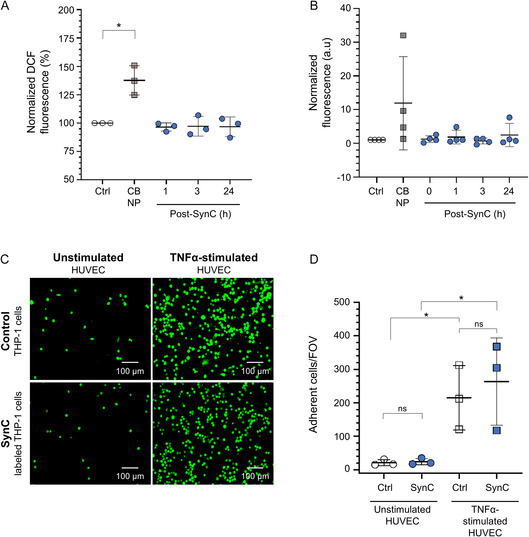
Cell‐functionality assessment post SynC labeling. (A) DCF assay for ROS quantification (*n* = 3). No altered DCF fluorescence 1, 3, and 24 h post SynC labeling compared to time‐matched controls, normalized to 100%. The significant increase in DCF fluorescence after exposure to CB NP validates assay sensitivity. (B) Caspase‐1 activity assay (*n* = 4) revealed no significant increase in cas‐1 activity after SynC labeling compared to time‐matched controls. CB NP‐treated cells showed a trend toward increased cas‐1 activity. (C) Representative images (20x magnification) of fluorescent THP‐1 cell adhesion to unstimulated and TNF*α*‐stimulated endothelial cells 3 h post SynC labeling. (D) Quantification of adherent cells per FOV (*n* = 3). No differences in the adhesion of SynC‐labeled and unlabeled THP‐1 cells to TNF*α*‐stimulated endothelial cells. (A–C) At each time point, the two groups were compared with one‐way ANOVA, followed by Tukey's multiple comparison test. * = *p* < 0.05. Data shown as mean ± SD. ANOVA = Analysis of variance; CB NP = carbon black nanoparticles; FOV = field of view; SD = standard deviation; TNFα, tumor necrosis factor alpha.

To facilitate MPI‐based cell tracking, it is essential to confirm that cell labeling itself does not impair cell function. To evaluate whether Synomag‐COOH (SynC) labeling affects cell physiology, we measured ROS levels in THP‐1 cells following 10 min SynC labeling. Intracellular ROS levels were quantified 1, 3, and 24 h postlabeling using a DCF‐based fluorescence assay. SynC‐labeled THP‐1 cells showed no significant increase in DCF fluorescence at any time point postlabeling. Upon SynC incubation, DCF fluorescence ranged from 96.50% (±2.93%) 1 h after labeling to 96.84% (±7.07%) 24 h post SynC labeling compared to time‐matched controls, normalized to 100% (Figure 1A). In contrast, exposure to carbon black nanoparticles (CB NP) significantly increased DCF fluorescence to 137.60% (±10.57%) compared to controls, confirming assay sensitivity. Our findings demonstrate that SynC labeling does not trigger ROS activation. In contrast to uncoated iron oxide nanoparticles, which have been reported to elevate intracellular ROS in endothelial cells, likely due to the release of free iron that drives ROS formation via the Fenton reaction [44, 45]. This suggests that the citrate coating of SynC limits the release of free iron upon internalization. The short labeling employed here could also prevent acute and delayed oxidative stress [46, 47]. Excessive ROS induction can impair cell viability and function. Thus, minimizing ROS induction is crucial for MPI‐based cell tracking. In MPI, accurate readouts rely on tracer retention and the physiological integrity of labeled cells [31, 32, 33].

Building on the ROS findings, we next assessed whether SynC labeling provokes inflammatory activation in THP‐1 cells. Certain metal and nonmetal nanoparticles were shown to activate the NLRP3 inflammasome upon internalization [48, 49]. To verify that SynC labeling does not trigger downstream inflammatory activation, we investigated inflammasome activation. Caspase‐1 (Cas1) activity was evaluated as a marker for inflammasome activation, immediately post SynC labeling and after 1h, 3, and 24 h (Figure 1B). Cas1 activity was unchanged immediately after SynC labeling compared to time‐matched controls. During the following hour, cas1 activity showed a transient, but statistically insignificant increase to 2.79 (±3.55) before returning to 0.84 (±0.91) and 2.50 (±2.99) after 3 and 24 h, respectively. In contrast, treatment with CB NP triggered an 11.85‐fold (±11.92) increase in cas1 activation. The absence of significant cas1 activation indicates that SynC does not elicit an immune response in THP‐1 cells. This is a key requisite for in vivo cell tracking, as cells with a preactivated inflammatory response could display altered migration behavior. These findings are consistent with previous reports showing that very small iron oxide nanoparticles (VSOP) do not significantly affect cytokine production and caspase3 activity [46, 50].

Preserved adhesion capacity of monocytes after nanoparticle labeling is critical for accurate immune cell tracking and inflammation mapping, as it ensures that immune cell labeling does not introduce artifacts in functional assays. Previous studies have shown that certain nanoparticles can affect the adhesion of monocytes to endothelial cells [51, 52]. To assess whether SynC labeling affects this key functional property, we evaluated the adherence of prior SynC‐labeled THP‐1 cells to human umbilical vein endothelial cells (HUVECs), which were either unstimulated or stimulated with tumor necrosis factor alpha (TNF*α*).

Under basal conditions, 21 (±7) unlabeled THP‐1 cells and 24 (±8) SynC‐labeled THP‐1 cells, 3 h post SynC labeling, adhered to endothelial cells within the field of view (FOV) (Figure 1C,D). Similar results were observed 24 h after SynC labeling (Figure S1, Supporting information (SI)). TNF*α* stimulation of endothelial cells significantly increased the adherence of THP‐1 cells, as expected. Importantly, this adhesion capacity was preserved upon SynC labeling of THP‐1 cells. We measured 263 (±106) SynC‐labeled THP‐1 cells compared to 215 (±78) unlabeled THP‐1 cells adhering to TNF*α*‐stimulated endothelial cells within the FOV at 3 h post SynC labeling (Figure 1C,D). The preserved adhesion capacity was verified 24 h after SynC labeling (Figure S1, SI). The high variability after TNF*α* stimulation reflects donor‐specific responses to TNF*α*, as the endothelial cells were derived from three different individuals. TNF*α* stimulation of endothelial cells induces the upregulation of the adhesion molecules E‐selectin, P‐selectin, ICAM‐1, and VCAM‐1. This results in enhanced monocyte adherence during inflammation [53, 54]. These results suggest that SynC labeling does not impair the interaction of THP‐1 cells with adhesion molecules on endothelial cells. This indicates that monocyte adhesion molecules remain accessible and functional without being masked by SynC particles [53, 55]. Overall, our findings support the suitability of SynC as a cell‐compatible MPI tracer, complementing prior observations that SynC labeling does not affect cell proliferation [43]. The absence of ROS induction, inflammatory activation, and altered adhesion to endothelial cells minimizes the risk of altered cell migration patterns, loss of labeled cells, or signal misinterpretation of MPI signals due to nanoparticle release upon cell death, enabling accurate immune cell tracking and inflammation mapping.

### 2.2 Magnetic Signatures of Internalized SynC Change during Longitudinal Measurements

**FIGURE 2:**
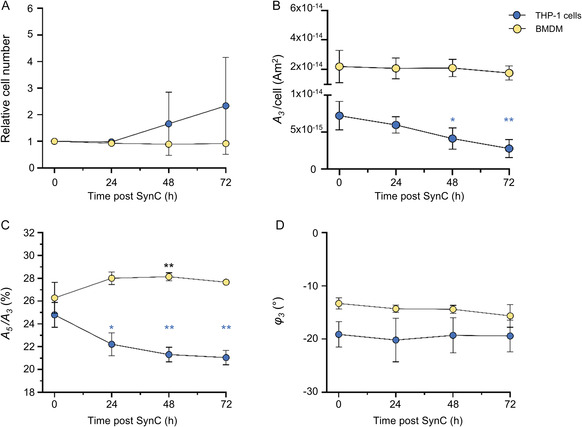
Magnetic parameters of SynC in dividing THP‐1 cells (blue) and nondividing macrophages (yellow) change during longitudinal measurements. Cells were labeled with SynC and analyzed with MPS over 72 h (*n* = 4, mean ± SD). (A) Relative cell numbers over 72 h. (B) The *A*_3_/*cell* signal decreased over 72 h in dividing THP‐1 cells and in BMDM. (C) The *A*_5_ */A*_3_ ratio decreases significantly after 24 h in THP‐1 cells and further declines in the following 48 h. In BMDM, the *A*_5_ */A*_3_ ratio signal increased significantly within the initial 48 h and declined subsequently. (D) The third harmonic phase remains relatively stable in THP‐1 cells and BMDM over 72 h. (B–D) Statistical analysis of longitudinal changes within each cell type was performed relative to the 0 h time point (*t*_0_). THP‐1 data were tested with one‐way ANOVA, followed by Tukey's multiple comparison test. BMDM data were compared with the Kruskal–Wallis test followed by Dunn's multiple comparison. * = *p* < 0.05, ** = *p* < 0.005. ANOVA = Analysis of variance; BMDM = bone marrow‐derived macrophages; MPS = magnetic particle spectroscopy; SD = standard deviation.

We next assessed whether intracellular processes influence the magnetic signature of internalized SynC. During longitudinal cell tracking, intracellular processing, lysosomal degradation, or cell proliferation could affect quantitative MPI. We used MPS to determine the magnetic signatures of SynC internalized by proliferating THP‐1 cells or nonproliferating bone marrow‐derived macrophages (BMDM) over 3 days. Data analyses were performed separately within each cell type to assess cell‐specific changes over time. MPS allows evaluating the dynamic magnetic signatures of a tracer without the complexity of MPI. We focused on the *A*_3_ signal intensity as a quantitative measure, combined with the *A*_5_ */A*_3_ ratio, and the phase of the third harmonic (*φ*_3_), which are sensitive to the nanoparticle environment, to indicate the MPI performance of the tracer [56] Over 72 h, the number of THP‐1 cells doubled from 0.8·10^6^ (±0.2·10^6^) to 1.6·10^6^ (±0.7·10^6^) cells (Figure 2A). During this time interval, the *A*_3_ signal per cell (*A*_3_ */cell*) significantly decreased from 7.23·10^−15^ Am^2^ (±1.67·10^−15^ Am^2^) directly after cell labeling to 2.79·10^−15^ Am^2^ (±1.06·10^−15^ Am^2^) after 72 h. The pronounced signal reduction in proliferating THP‐1 cells likely arises from a combination of tracer dilution during cell division and nanoparticle degradation. During proliferation, daughter cells inherit a fraction of the internalized tracer, resulting in a progressive decrease of the magnetic signal per cell. This is a well‐known issue during longitudinal cell tracking studies. Current mitigation strategies include high initial labeling loads or mathematically modeling cell proliferation during data interpretation [57]. In addition to cell division‐induced dilution, proliferation‐independent processes, such as lysosomal nanoparticle degradation, are expected to further contribute to *A*_3_ signal loss [41]. Consistent with this interpretation, a progressive decrease in the *A*_3_ */cell* was also observed in nondividing BMDM, where the *A*_3_ */cell* declined from 2.20·10^−14^ Am^2^ (±9.53·10^−15^ Am^2^) right after labeling to 1.76·10^−14^ Am^2^ (±4.13·10^−15^ Am^2^) after 72 h (Figure 2B). This observation revealed *A*_3_ signal loss even in the absence of cell division, indicating a contribution from proliferation‐independent processes. A likely mechanism is lysosomal nanoparticle degradation, as the acidic environment in lysosomes can destabilize SynC particles and promote nanoparticle disassembly, resulting in reduced magnetic anisotropy and *A*_3_ signal intensity [23, 58]. Together, this indicates a balance between short‐term intracellular signal stability of SynC and progressive biodegradation. This is a requirement for in vivo application as it offers reliable cell tracking within the relevant observation window while eventual ensuring eventual nanoparticle disassembly. Nonetheless, progressive nanoparticle degradation and proliferation‐induced dilution contribute to reduced MPI performance over time and must be considered when analyzing longitudinal measurements. Interestingly, BMDM exhibited a higher initial *A*_3_ signal compared to THP‐1 cells, suggesting greater SynC uptake efficiency or extracellular nanoparticle binding during the 10 minute labeling period.

In THP‐1 cells, the concentration‐independent *A*_5_ */A*_3_ ratio declined significantly within the first 24 h, dropping from 24.80% (±0.94%) to 22.21% (±0.82%), and then stabilizing at around 21.04% (±0.55%) after 72 h (Figure 2C). This behavior suggests that rapid structural alterations occur during initial lysosomal processing, which equalize once particles are confined within mature phagolysosomes. The rapid *A*_5_/*A*_3_ decline, 24 h after nanoparticle uptake, was previously observed for smaller VSOP nanoparticles within THP‐1 cells and macrophages. The same trend but with opposite direction was observed in BMDM, where the *A*_5_ */A*_3_ ratio significantly increased within the first 48 h from 26.28% (±1.18%) to 28.14% (±0.31%), before stabilizing around 27.66% (±0.04%) after 72 h (Figure 2C). The higher *A*_5_ */A*_3_ baseline in BMDM at 26.28% compared to 24.58% in THP‐1 cells may reflect stronger initial aggregation at the macrophage membrane. The *A*_5_/*A*_3_ rise recorded from SynC internalized in BMDM suggests further aggregation or structural reorganization within endolysosomal compartments, potentially enhancing MPI performance at intermediate time points. Slower SynC processing in macrophages compared to monocytes could explain the delayed *A*_5_/*A*_3_ ratio decline. From an MPI perspective, these distinct *A*_5_/*A*_3_ ratios of SynC after internalization represent an opportunity for imaging SynC degradation based on degradation‐specific magnetic signatures, as previously hypothesized [37].

The phase of the third harmonic remained relatively stable in dividing THP‐1 cells and BMDM across 72 h. In THP‐1 cells, the *φ*_3_ measured −19.12° (±2.08°), directly after cell labeling. It decreased slightly to −20.20° (±3.35°) after 24 h, before returning to −19.41° (±2.58°) after 72 h (Figure 2D). This indicates that cell division is not reflected in the parameter *φ*_3_. In BMDM, *φ*_3_ also decreased slightly from −13.30° (±0.90°) right after cell loading to −15.65° (±1.86°) after 72 h. The stability of the *φ*_3_ diverges from prior reports of phase shift reductions during nanoparticle degradation in lysosomes [60]. The discrepancy likely reflects the dominance of Néel relaxation in internalized SynC, with a small core size of 13.5 nm, where Brownian relaxation is negligible [41, 43, 61, 62]. Moderate degradation of SynCs multicore may not reduce the core size below a critical threshold to alter Néel relaxation times appreciably [41, 61, 62].

In summary, the *A*_3_ signal and *A*_5_ */A*_3_ ratio of SynC internalized by dividing THP‐1 cells change significantly over 72 h, reflecting the combined effects of cell proliferation and intracellular processing. In contrast, these magnetic parameters were more stable in nondividing BMDM, though intracellular processing remained evident in the *A*_3_ signal decrease and significant increase of the *A*_5_ */A*_3_ ratio 48 h post SynC internalization. These results underscore the importance of considering cell type and intracellular nanoparticle degradation dynamics when designing long‐term MPI‐based cell tracking studies. The absence of labeling‐induced oxidative stress and inflammasome activation, demonstrated in the prior section, supports that the observed magnetic signature changes arise from cell physiological processes rather than labeling artifacts. Overall, these findings indicate that the magnetic signatures of internalized SynC evolve during intracellular processing, suggesting that MPI could be used to noninvasively image and monitor dynamic intracellular processes over time.

### 2.3 Changes in the Magnetic Signature of Internalized SynC Caused by Extracellular Oxidative Stress Can Be Visualized with Color Magnetic Particle Imaging

**FIGURE 3:**
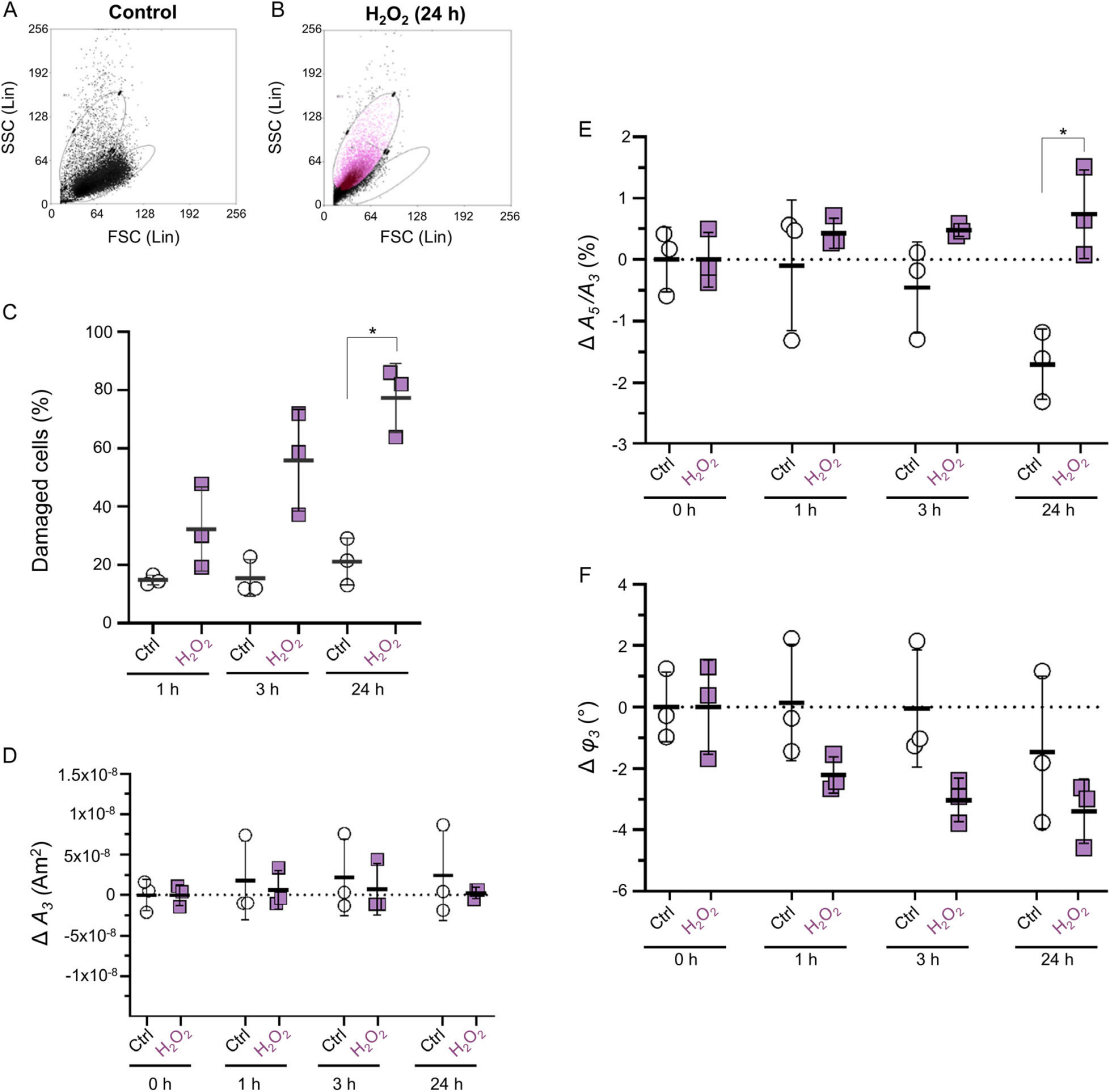
Effect of extracellular oxidative stress on SynC‐labeled THP‐1 cells and magnetic parameters of cell‐associated SynC. SynC‐labeled THP‐1 cells were exposed to H_2_O_2_ to assess the effect of extracellular oxidative stress on cellular morphology and the magnetic parameters of SynC. (A–C) Flow cytometry (*n* = 3) assesses oxidative cell damage by FSC and SSC. Two distinct populations were defined and gated. (A) FSC (size) and SSC (granularity) of SynC‐labeled control cells. (B) H_2_O_2_ treatment of previously SynC‐labeled cells results in a shift to lower FSC (decreased cell size) and higher SSC (increased granularity), indicative of cell damage. (C) Quantification showed a significant increase in the proportion of damaged cells after 24 h of H_2_O_2_ treatment. (D–F) MPS measurements of untreated and H_2_O_2_‐treated SynC‐labeled THP‐1 cells for 1, 3, and 24 h (*n* = 3). Differences are shown relative to *t*_0_. (D) *A*_3_ signal amplitude remains stable in control and damaged cells over time, indicating that SynC particles remain cell associated upon oxidative damage. (E) The *A*_5_/*A*_3_ ratio declined in control cells over time, and increased in H_2_O_2_‐treated cells, resulting in a significant difference after 24 h. (F) Phase shift decreased modestly in both groups, with a greater reduction observed in the H_2_O_2_‐treated sample. (C–F) Time‐point matched comparisons between the two groups were conducted using one‐way ANOVA, followed by Tukey's multiple comparison test. * = *p* < 0.05. Data shown as mean ± SD. ANOVA = Analysis of variance, FSC = forward scatter; SSC = side scatter; SD = standard deviation.

We next investigated whether the magnetic parameters of internalized SynC change upon extracellular oxidative cell damage. This is particularly relevant for MPI‐based immune cell tracking, as immune cells experience oxidative environments at sites of inflammation. At inflammatory sites, activated immune cells release high levels of ROS, such as H_2_O_2_, during the respiratory burst triggered by phagocytosis. At physiological levels, ROS act as signaling molecules that help maintain tissue homeostasis. However, under pathological conditions, excessive ROS accumulation oxidatively damages cellular components, including DNA, proteins, and lipids. Lipid peroxidation in particular compromises the integrity of cell membranes. When cellular damage becomes extensive, it can ultimately drive cell death through apoptosis or necrosis [42, 63, 64, 65]. To model this oxidative environment, SynC‐labeled THP‐1 cells were exposed to H_2_O_2_ for 1 h, 3 h, or 24 h, allowing us to assess how extracellular oxidative stress influences the magnetic parameters of SynC over time. Flow cytometry confirmed progressive cell damage in the H_2_O_2_‐treated sample, evident in the reduced cell size and increased granularity [66] (Figure 3A,B). In the untreated control, the proportion of damaged cells ranged from 14.58% (±1.27%) after 1 hr to 21.27% (±6.59%) after 24 h. In the H_2_O_2_‐treated sample, this significantly increased from 34.38% (±12.39%) after 1 h to 77.07% (±9.30%) after 24 h H_2_O_2_ treatment (Figure 3C). Thus, the H_2_O_2_ conditions applied here caused measurable cell damage, evident in altered cell size and granularity, providing a model for the oxidative microenvironment found in inflamed tissue. Both treated and control samples contained a mixed population of viable and damaged cells, which we considered when interpreting the magnetic readouts.

Considering the characteristic MPS parameters, we found that the *A*_3_ amplitude remained stable across all time points and treatment groups. Even after 24 h of H_2_O_2_ treatment, which damaged most cells, the *A*_3_ signal intensity did not significantly differ compared to untreated controls (Figure 3D). This indicates that SynC remains cell‐associated despite oxidative damage to cell membranes, which could have potentially promoted nanoparticle release. The *A*_5_/*A*_3_ ratio diverged markedly between untreated and H_2_O_2_‐treated cells over time. Over 24 h, controls again showed an *A*_5_/*A*_3_ ratio decline by 1.70% (±0.28%), whereas H_2_O_2_‐treated cells exhibited an increase by 0.74% (±0.81%) (Figure 3E). The *φ*_3_ of SynC trended downward in H_2_O_2_‐treated cells compared to untreated controls. While SynC in control cells only exhibited a 1.47° (±1.46°) phase shift over 24 h, SynC internalized in H_2_O_2_‐treated cells showed a cumulative *φ*_3_ change of 2.21°, 3.03°, and 3.39° after 1, 3, and 24 h of H_2_O_2_ treatment (Figure 3F). Although high interexperimental variability in the control samples limited statistical significance, the pattern is consistent with phase shifts reported during nanoparticle degradation [41, 60]. In summary, the MPS measurements revealed a 2.44% divergence in the *A*_5_ */A*_3_ ratio and a larger *φ*_3_ shift of SynC internalized by cells exposed to extracellular oxidative stress compared to untreated control cells. Thus, external oxidative stress propagates into intracellular environments in ways that measurably affect the magnetic parameters of nanoparticles already internalized within cells. This underscores the sensitivity of magnetic nanoparticle dynamics to subtle changes in cell physiology. Flow cytometry confirmed that H_2_O_2_ treatment induced cell shrinkage and increased granularity, which could influence intracellular nanoparticle aggregation or agglomeration. Additional factors that may affect the magnetic parameters of internalized SynC include disrupted intracellular compartmentalization or altered intracellular viscosity upon oxidative damage [67, 68]. The oxidative environment induced by H_2_O_2_ could also directly destabilize SynC by oxidizing or displacing the citrate coating, thereby increasing nanoparticle aggregation [69, 70].

Key differences in MPS parameters of SynC internalized by viable and oxidatively damaged cells indicate distinct magnetic signatures of SynC internalized by these two cell physiological states. To test whether these can be distinguished with color MPI, we first introduce the determination of system matrices that exclusively contain viable ($\bar{S_{v}}$) or oxidatively damaged cells ($\bar{S_{d}}$). These system matrices were mathematically derived from samples containing mixed cell populations ($\bar{S_{1}}$, $\bar{S_{2}})$, based on the results of the 24 h flow cytometry measurements. The control sample ($\bar{S_{1}}$) consisted of 78.73% viable and 21.27% oxidatively damaged cells, whereas the H_2_O_2_‐treated reference $\left(\bar{S_{2}}\right)$ contained 22.93% viable and 77.07% oxidatively damaged cells. The linear system of equations (Equation (1)) was established, enabling the derivation of the pure system matrices ($\bar{S_{v}}$, $\bar{S_{d}}$)

(1) $$\begin{array}{c} \bar{S_{1}}=0.7873\cdot \bar{S_{v}}+0.2127\cdot \bar{S_{d}}\\ \bar{S_{2}}=0.2293\cdot \bar{S_{v}}+0.7707\cdot \bar{S_{d}} \end{array}$$

**FIGURE 4:**
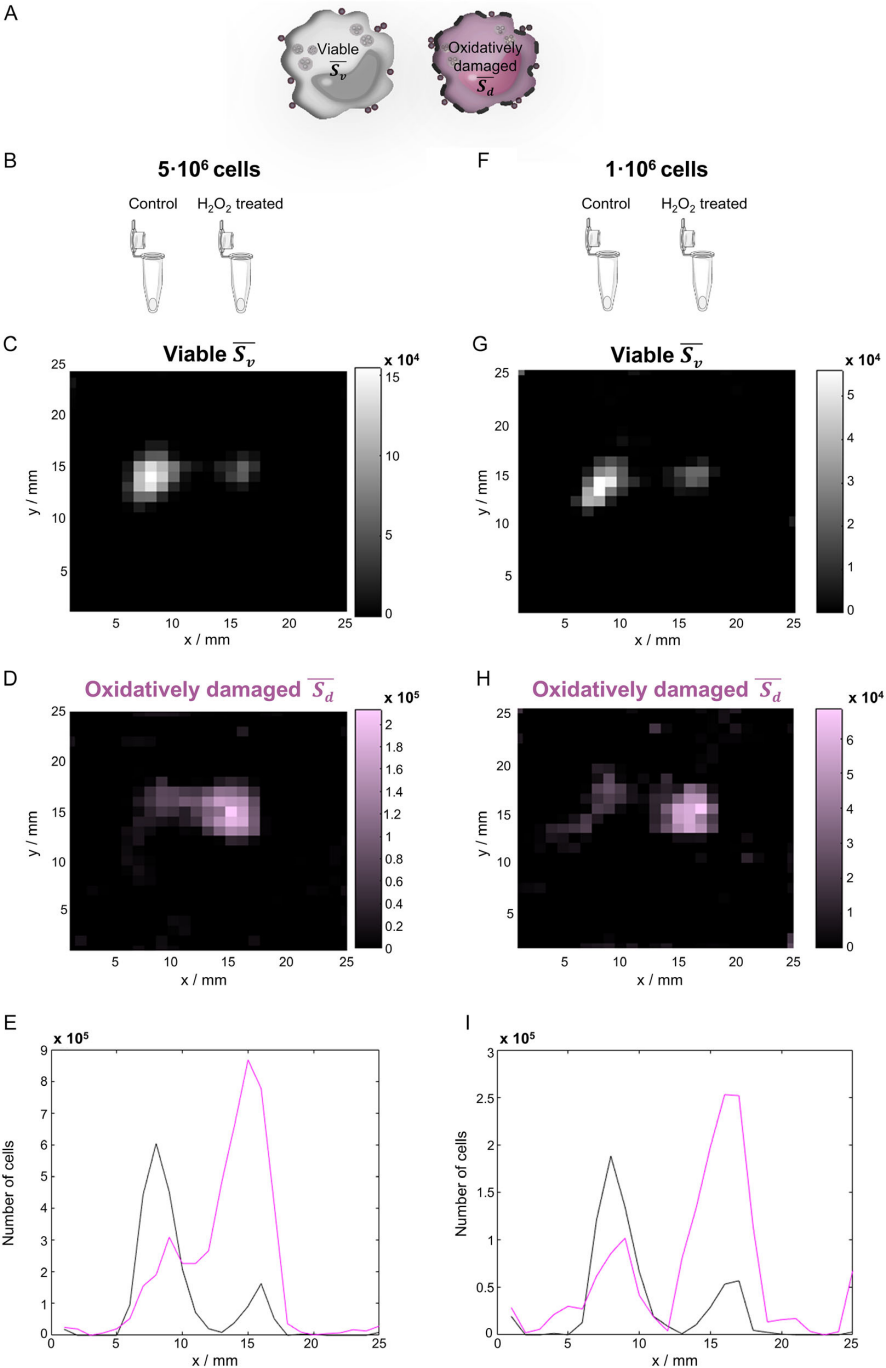
Color MPI to distinguish viable and oxidatively damaged SynC‐labeled THP‐1 cells. (A) Schematic of cell types used to acquire system matrices. (B–E) MPI measurement of two simultaneously measured samples, each containing 5·10^6^ SynC‐labeled THP‐1 cells. (B) Side‐by‐side sample position of control samples (left) and H_2_O_2_‐treated samples (right). (C and D) MPI images reconstructed using the (C) viable system matrix ($\bar{S_{v}}$) or (D) oxidatively damaged system matrix ($\bar{S_{d}}$). (H) Cell number distributions obtained with $\bar{S_{v}}$ (black) and $\bar{S_{d}}$ (pink), demonstrating a clear differentiation between viable and oxidatively damaged cells in mixed populations. (F–I) MPI measurement of two simultaneously measured samples, each containing 1·10^6^ SynC‐labeled THP‐1 cells. MPI = Magnetic particle imaging.

During the measurements, a control sample and an H_2_O_2_‐treated sample, each containing 5·10^6^ THP‐1 cells, were placed side by side and measured simultaneously (Figure 4B). When both samples were reconstructed using $\bar{S_{v}}$, signals were predominantly localized in the control sample, whereas $\bar{S_{d}}$ reconstructed more cells in the H_2_O_2_‐treated sample (Figure 4C–E). Notably, $\bar{S_{d}}$ also identified a subpopulation of damaged cells in the control sample. This demonstrates the sensitivity of color MPI to resolve heterogeneous mixtures of viable and oxidatively damaged cells. Repeated experiments verified the capacity to reconstruct variable proportions of viable and oxidatively damaged cells across independent experiments (Figure S2, SI). Even at reduced cell numbers of 1·10^6^ cells per sample, the distinct magnetic signatures of viable and oxidatively damaged cells remained clearly discernible (Figures 4F–I and S3, SI). These results showed that SynC internalized in viable and oxidatively damaged cells exhibits separable magnetic signatures that can be distinctly imaged with color MPI. The MPS measurements revealed an increased *A*_5_ */A*_3_ ratio and greater *φ*_3_ shifts of SynC within oxidatively damaged cells. These changes may arise from SynC aggregation during apoptotic cell shrinkage, altered intracellular viscosity, and direct destabilization of SynC by H_2_O_2_. Because SynC signals are likely dominated by Néel relaxation, tracers dominated by Brownian relaxation are expected to show even greater sensitivity to viscosity‐dependent effects [41, 71].

Building on the separation capability, the quantitative performance of the functional color MPI approach was evaluated. Combining $\bar{S_{v}}$ and $\bar{S_{d}}$ during image reconstruction enabled reconstruction of 10·10^6^ (±2.1·10^6^) cells using the total reconstruction method across both samples, matching the expected total of 10·10^6^ cells. The segmented reconstruction approach quantified 8.6·10^6^ (±2.1·10^6^) cells (Figure S4, SI). These results confirm that functional color MPI can not only distinguish cellular states but also quantitatively capture the total labeled cell population with high accuracy. The quantitative MPI signal increased with higher numbers of labeled cells. In principle, higher iron loading per cell could further enhance MPI performance. However, the biological tolerance and reproducibility of SynC loading define the practical upper limit of particle loading in THP‐1 cells, rather than MPI performance, which supports the application of the selected labeling protocol for robust and physiologically meaningful measurements [43].

To our knowledge, this represents the first demonstration that color MPI can distinguish viable from oxidatively damaged monocytes within mixed populations by leveraging system matrices tuned to magnetic signatures of intracellular nanoparticles. This proof of concept demonstrates that color MPI can image cell physiology beyond mere cell localization. If validated in vivo, this approach would provide a noninvasive method to monitor cell viability during cell tracking, thereby adding a functional dimension to color MPI. This capability opens new opportunities for tracking the fate of immune cells and therapeutic efficacy during immunotherapy.

## 3 Conclusion

We conclude that MPI can distinguish cell physiology‐dependent magnetic signatures, thereby extending its capability beyond spatial localization to functional imaging of cellular physiology. Exposure of SynC‐labeled cells to extracellular oxidative stress resulted in distinct magnetic signatures of internalized SynC. This enabled color MPI to distinguish viable from oxidatively damaged cells within heterogeneous cell populations. Such functional color MPI requires precise knowledge of the influence of particle labeling on the cellular system. The cellular compatibility of each tracer must be carefully evaluated for each cell type to facilitate functional color MPI. This work provides proof of concept for MPI‐based functional imaging of cellular states. Once validated in vivo, functional color MPI could enable noninvasive monitoring of dynamic cell physiology and the assessment of therapeutic efficacy, for example, by tracking the viability of injected cells during regenerative and immunotherapy treatments.

## 4 Methods

### 4.1 Cell Culture

The human acute monocytic leukemia THP‐1 cell line (ACC 16, DSZM) was used for all in vitro experiments unless specified otherwise. Cells were maintained in complete RPMI 1640 medium (cRPMI, Gibco Life Technologies) supplemented with 10% fetal calf serum (FCS, Biochrom), penicillin (100 U mL^−1^, Invitrogen), and streptomycin (100 µg per mL streptomycin, Invitrogen). Medium was refreshed every 2–3 days or when the cell density exceeded 5·10^5^ cells mL^−1^. All cell types were incubated at 37°C in a humid atmosphere containing 5% CO_2_ in 75 cm^2^ cell culture flasks. Cell numbers were determined by counting with a Fuchs–Rosenthal counting chamber.

To investigate the adhesion capacity of labeled THP‐1 cells to endothelial cells under basal and inflammatory conditions, we used HUVECs. Primary HUVECs were isolated from different umbilical cord donors following a previously described protocol [72]. The isolation and handling of HUVECs were approved by Charité—Universitätsmedizin Berlin Ethics committee (license number: EA2/056/24) and conducted in accordance with institutional guidelines and the principles of the Declaration of Helsinki. All participants gave their consent to participate, and their anonymity was maintained. HUVECs were cultured in MCDB 131 medium (Gibco Life Technologies) supplemented with 2% FCS, penicillin (100 U mL^1^), streptomycin (100 µg mL^−1^), heparin (5 U mL^−1^, Merck), basic fibroblast growth factor (0.5 µL mL^−1^, Biomol), epidermal growth factor (0.1 ng mL^−1^, Biomol), hydrocortisone (1 µg mL^−1^, Sigma‐Aldrich), L‐glutamine (10 µL mL^−1^, Thermo Fisher Scientific), and endothelial cell growth supplement (4 µL mL^−1^, Promocell). HUVECs were used for experiments until passage five.

To investigate the magnetic parameters of internalized SynC in nondividing cells, we used BMDM. The cells were isolated from four adult wild‐type C57/BL6 mice (two female and two male). Animal procedures were approved by Charité—Universitätsmedizin Berlin in accordance with the national Animal Welfare Act and international guidelines (license number = T‐CH 0015/23). The mice were housed under pathogen‐free conditions and had *ad libitum* access to food and water, with a 12 h dark/light cycle. The mice were anesthetized with isoflurane using the drop jar method and sacrificed via cervical dislocation under deep anesthesia, confirmed by loss of pedal reflex. BMDM were isolated as previously described [73]. Cells were seeded (1·10^7^ cells) into a 10 cm^2^ Petri dish (Falcon, Sigma‐Aldrich) 4–5 days before the experiment. BMDM were cultured in cRPMI supplemented with 10% L929 cell‐conditioned medium (LCCM). LCCM was collected from L929 cells as described in earlier studies [74]. In short, LCCM was collected from 1.2·10^5^ L929 cells seeded in a 75 cm^2^ flask and grown for 7 days in RPMI 1640 medium supplemented with 7.5% FCS, 100 U mL^−1^ penicillin, and 100 mg mL^−1^ streptomycin.

### 4.2 Nanoparticle Cell Labeling

Synomag‐COOH (SynC), a citrate‐coated iron oxide nanoparticle system, was obtained from micromod Partikeltechnologie GmbH (article no. 103‐02‐301). It was previously determined that SynC nanoparticles have a hydrodynamic diameter of 23.4 ± 6.2 nm, a core size of 13.5 ± 0.2 nm, and a negative surface charge of −13.8 ± 2.1 mV [43]. 1·10^6^ cells were incubated with c(Fe) = 0.5 mmol L^−1^ SynC for 10 min in 1 mL phosphate‐buffered saline (PBS) at 37°C with 5% CO_2_. This concentration was selected based on a prior systematic optimization study, which identified it as providing robust iron loading (≈5 pg per cell, corresponding to 18% of the administered 27.9 µg Fe) while maintaining cell viability [43]. SynC labeling was performed in PBS without serum proteins to exclude protein–nanoparticle interactions and protein corona formation before nanoparticle–cell interactions and cellular uptake. Following labeling, cells were washed twice with PBS to remove unbound particles. Thus, all measurements reflected predominantly internalized or strongly cell‐bound SynC [43]. The intracellular localization of SynC nanoparticles was previously confirmed using TEM after applying the 10 min labeling protocol [23].

CB NP (ROTInanoMETIC ≥99%, 10–20 nm, Carl Roth), a nonmagnetic nanoparticle system, were used as a positive control that is known to induce an inflammatory and oxidative stress response within cells [75]. A stock solution was prepared by dispersing 6 mg of CB NP in 10 mL dH_2_O. The suspension was sonicated for 20 min at 50% amplitude with 0.5 s impulses (Bandelin Sonopuls HP 70). Before each experiment, sonication was repeated with the same settings for 15 min. THP‐1 cells were incubated with 60 µg mL^−1^ CB NP for 24 h in cRPMI medium to achieve sufficient induction of oxidative stress and inflammation [76].

### 4.3 DCF Assay

To assess whether SynC labeling induces oxidative stress, we performed a 2′,7′‐dichlorfluorescein‐diacetate (H_2_DCFDA; DCF) assay. During the assay, H_2_DCFDA reacts with ROS to fluorescent DCF [77]. DCF levels were normalized to time‐matched controls to account for circadian fluctuations [78]. THP‐1 cells were washed with PBS and labeled with SynC for 10 min. After subsequent washing with PBS, cells were resuspended in cRPMI medium. Between all washing steps, the samples were centrifuged for 2 min at 1000 rpm unless stated otherwise. The samples were either processed immediately or incubated for 1, 3, or 24 h at 37°C after SynC labeling. After incubation, the samples were centrifuged for 5 min at 1000 rpm, washed with PBS, and resuspended in RPMI without supplements containing 10 µL mL^−1^ H_2_DCFDA (PromoCell). The reaction proceeded for 30 min at 37°C in the dark. Cell numbers were normalized to 100,000 cells per sample. After a final PBS wash, 100 µL of the sample was transferred to a 96‐well plate, and fluorescence was measured at 529 nm (excitation: 488 nm) using a microplate reader (Molecular Devices Spectra Max Gemini EM). Untreated and CB NP‐treated cells served as negative and positive controls, respectively. The mean of three independent experiments with two or three technical replicates measured per condition is shown.

### 4.4 Caspase‐1 Assay

To examine whether SynC nanoparticles activate the inflammasome, we performed a fluorometric caspase‐1 assay. THP‐1 cells (1·10^6^ cells mL^−1^) were incubated with SynC for 10 min, followed by washing with PBS. Samples were collected immediately after SynC incubation and 1, 3, or 24 hr later. Untreated THP‐1 cells were used as a negative control, and CB NP‐treated cells were used as a positive control. Samples were lysed on ice for 10 min with 250 µL of lysis buffer containing 10 mmol L^−1^ Tris (Promega), 10 mmol L^−1^ NaH_2_PO_4_/Na_2_HPO_4_ (Merck), 130 mmol L^−1^ NaCl (Carl Roth), and 1% Triton X‐100 (Sigma‐Aldrich). Lysates were centrifuged at 4°C for 5 min at 13 200 rpm (Centrifuge 5415R, Eppendorf). Two hundred µL of the supernatant was mixed with 200 µL reaction buffer. The reaction buffer contained 40 mmol L^−1^ HEPES (pH 7.5, Merck), 40% Glycerol (Sigma‐Aldrich), freshly added 8 mmol L^−1^ DTT (Sigma‐Aldrich), and 4 µL fluorogenic caspase‐1 substrate (5 mg mL^−1^ in DMSO, SCP0073, Sigma‐Aldrich). One hundred µL of the sample was transferred into a black 96‐well plate (Greiner Bio One) and incubated for 2 h at 37°C. The fluorescence was measured at an excitation of 380 nm and an emission wavelength of 460 nm using a plate reader. The background fluorescence was measured after 5 min of incubation and subtracted from the result. Four independent experiments, each with three technical replicates per condition, were performed.

### 4.5 Quantification of THP‐1 Adherence to Endothelial Cells

To determine whether SynC labeling affects the ability of monocytes to adhere to endothelial cells under basal and inflammatory conditions, an adhesion assay was performed. THP‐1 cells were incubated with SynC, washed, and fluorescently labeled with 5 µg mL^−1^ calceinAM (Invitrogen) for 60 min in RPMI medium supplemented with 1% FCS, 100 U mL^−1^ penicillin, and 100 µg mL^−1^ streptomycin. Fluorescently labeled cells were resuspended in cRPMI medium, and either SynC‐labeled or control THP‐1 cells were added to a monolayer of endothelial cells (HUVEC). Twenty‐four h before performing the assay, 100,000 HUVECs were seeded into an 8‐well slide (ibidi). To mimic inflammatory conditions, HUVECs were stimulated with 5 ng mL^−1^ recombinant human TNF*α* (Promocell) for 4 h. Both TNF*α*‐stimulated HUVECs and unstimulated HUVECs were then incubated with fluorescently labeled THP‐1 cells for 30 min at 37°C. Nonadherent THP‐1 cells were removed by gently washing with PBS. The adherent cells were visualized and quantified using a fluorescent microscope (BZX‐810, Keyence). The experimental design included controls without SynC‐labeling and TNF*α* stimulation and setups with only one of the treatments. Three images were taken for each experimental condition at 20x magnification with equal imaging parameters. The number of adherent cells was determined using ImageJ (NIH) [79]. The images were first converted to 8‐bit grayscale and then to binary masks. To separate adjacent cells, the watershed algorithm was applied. Adherent cells were counted by applying the analyze‐particles function with a minimal particle size of 0.02 inch^2^. Particles touching the image borders were excluded from analysis. The means of three individual experiments using three different HUVEC donors are shown.

### 4.6 Magnetic Particle Spectroscopy

MPS measurements were conducted with an MPS‐3 spectrometer (Bruker). Samples were measured at an excitation amplitude of *B* = 12 mT and a frequency of *f*_0 _= 25 kHz. MPS measurements were used to assess the MPI performance of SynC‐labeled cells. The evaluation was conducted based on three characteristic magnetic parameters: the third harmonic amplitude (*A*_3_), the ratio of the fifth and third harmonic (*A*_5_ */A*_3_), and the phase of the third harmonic (*φ*_3_). The intensity of the *A*_3_ signal is directly proportional to the number of nanoparticles. The *A*_5_ */A*_3_ ratio and the *φ*_3_ are independent of the number of nanoparticles, sensitive to the nanoparticle microenvironment, and informative for MPI image reconstruction. [56, 80, 81] The *φ*_3_ indicates the capability of the nanoparticle moments to follow the magnetic excitation field and variations are caused by changes in the nanoparticle environment [82]. The *A*_5_ */A*_3_ ratio of SynC has been demonstrated to undergo substantial alterations upon interaction with cells [59]. To investigate the effects of cell division and particle degradation on the magnetic parameters of internalized SynC, longitudinal MPS measurements were performed in proliferating THP‐1 cells and nondividing BMDM. Measurements were recorded immediately after SynC cell labeling (*t*_0_) and after 24, 48, and 72 h. At each time point, an aliquot of 1 mL was taken from the flask containing SynC‐labeled cells and processed into cell pellets by centrifugation at 1000 rpm and measured with MPS in PCR tubes. All MPS parameters were analyzed as changes compared to the *t*_0_ baseline within each cell type. Four independent experiments, each with triplicate measurements per time point, were conducted.

### 4.7 Cell Damage Induction and Quantification

To evaluate the effect of extracellular oxidative damage on the magnetic parameters of internalized SynC, cell damage was induced using hydrogen peroxide (H_2_O_2_) (Merck). SynC‐labeled THP‐1 cells (1·10^6^) were resuspended in PBS and treated with 2% H_2_O_2_ for 1, 3, or 24 h at 37°C. Control samples received no H_2_O_2_ and were incubated in cRPMI medium for the same duration after SynC labeling. After incubation, cell viability was assessed using flow cytometry (Cyan ADP flow cytometer, Beckman Coulter). Cells with lower forward scatter (FSC) and higher side scatter (SSC) were classified as damaged, indicating a smaller size and higher granularity [66]. Three independent experiments, each with technical duplicates or triplicates, were performed for MPS and flow cytometry. For MPS measurements, 1 mL of the cell suspension was collected, transferred into an NMR glass tube (Bruker 180 NMR PC), and measured using the settings described in section 4.6.

### 4.8 Color Magnetic Particle Imaging to Visualize Cell Physiology

To evaluate whether MPI can distinguish viable from oxidatively damaged SynC‐labeled cells, color MPI was performed. Color MPI enables the distinction of magnetic signatures within a single acquisition and assigns them distinct colors. Each color channel incorporates a characteristic magnetic signature, for which a specific system matrix must be acquired as a reference for the magnetic behavior of that color [38, 39]. In this study, the color channels were not assigned to different magnetic nanoparticle systems but rather to distinct cell physiologies. This enabled visualization of changes in cell physiology that influence the magnetic signature of internalized nanoparticles. This approach is referred to as functional color MPI in this study. Cellular physiology was validated using flow cytometric assessment of cell size and granularity.

MPI was conducted using a preclinical MPI scanner (Bruker MPI 25/50 FF, GER) based on the field‐free point (FFP) approach and equipped with an additional separate mouse coil (Bruker) to increase sensitivity. The FFP is generated by a static magnetic field gradient (0.75/0.75/1.5 T/m in x/y/z‐direction) and superimposed by oscillating magnetic drive fields with field amplitudes of 12/12/12 mT. The system matrices served as a calibration dataset exploiting different magnetic signatures of the nanoparticle system caused by different cell states [83]. The system matrices were recorded using 100 averages, and a subsequent background measurement was subtracted. The measurements were performed in a FoV of 25 × 25 × 13 voxels with a voxel size of 1 × 1 × 1 mm^3^. The same parameters were used to record the experiment measurements. Two separate system matrices ($\bar{S}$) were recorded of samples containing 5·10^6^ SynC‐labeled THP‐1 cells either untreated for 24 h ($\bar{S_{1}}$) or treated with H_2_O_2_ for 24 h ($\bar{S_{2}}$). Naturally, both biological states of viable and damaged cells are always present concurrently for a given cell sample. To enable clear differentiation of these two cellular states, the pure system matrices of exclusively viable cells ($\bar{S_{v}}$) or oxidatively damage cells ($\bar{S_{d}}$) are required. Thus, the measured system matrices $\bar{S_{1}}$ and $\bar{S_{2}}$ are described as a mixture of the pure system matrices $\bar{S_{v}}$ and $\bar{S_{d}}$ and the resulting equation system Equation (1) is solved for $\bar{S_{v}}$ and $\bar{S_{d}}$. Three‐dimensional image reconstructions were performed by applying the system matrices to untreated control samples (*c*_v_) and H_2_O_2_‐treated samples (*c*_d_) after SynC labeling. Image reconstructions were performed by solving the following inverse problem by least squares methods using the Kaczmarz algorithm with Tikhonov regularization (regularization parameter $\lambda =\lambda_{r}\cdot \lambda_{0}$) [84].

(2) $${\left\|\left[\bar{S_{v}}\bar{S_{d}}\right]\left[\begin{array}{c} c_{v}\\ c_{d} \end{array}\right]-\bar{u}\right\|}^{2}+\lambda \left\|\left[\begin{array}{c} c_{v}\\ c_{d} \end{array}\right]\right\|\to \min$$

The spectral pattern of the MPI signal ($\bar{u}$) depends on the environmental conditions of SynC in viable and oxidatively damaged cells, and the spatial distribution within the FoV. In the experimental setting, the two samples containing untreated and H_2_O_2_‐treated cells were placed side by side and imaged simultaneously as two point sources. To increase reconstruction accuracy, background subtraction was applied. We used a regularization parameter *λ*_r_ = 10^−3^ and 20,000 Kaczmarz iterations to reconstruct images by solving Equation (2) [38, 85, 86]. The sum of the voxels for image reconstruction was determined based on two different approaches. Using the total approach, the sum of all voxels above zero was added. The segmented approach identified the voxel with the highest content. The contents of the adjacent voxels were successively summed, if the intensity of the voxels exceeded 10% of the maximum intensity, and iteratively added up the contents of the adjacent voxels as long as the intensity of the voxels was more than 10% of the maximum [43]. Cell numbers were estimated based on these two approaches using the system matrix as a reference. We performed measurements of 5·10^6^ SynC‐labeled THP‐1 cells, either untreated or treated with H_2_O_2_ for 24 h, using three separate samples prepared on different days. We also measured three further samples, containing 1·10^6^ SynC‐labeled THP‐1 cells, either untreated or treated with H_2_O_2_ for 24 h.

### 4.9 Data Analysis and Visualization

Graphs were generated using GraphPad Prism version 8 (*Software Inc.*)*.* The graphical abstract and schematic depictions were created with Inkscape (Inkscape Project Contributors 2025, version 1.4.21.0, https://inkscape.org/). The microtube illustration (Figure 4A) was obtained from Servier Medical Art (https://smart.servier.com/), licensed under CC BY 4.0 (https://creativecommons.org/licenses/by/4.0/).

### 4.10 Statistical Analysis

Statistical analysis was conducted with GraphPad Prism version 8 (Software Inc.). The results are presented as mean ± standard deviation (SD), with each data point including duplicate or triplicate measurements. A total of three to four independent experiments were conducted. All data points were included in the final analysis. Statistical significance was defined as *p* < 0.05. The data were tested for normal distribution using the Shapiro–Wilk test. Normally distributed data were tested with one‐way ANOVA (analysis of variance) followed either by Tukey's or Dunnett's post hoc test, depending on the comparison. When the data did not meet the assumptions of normality, we used the nonparametric Kruskal–Wallis test with Dunn's multiple comparison.

## Supporting Information

Additional SI can be found online in the Supporting Information section. **Supporting Fig. S1:** Adhesion of THP‐1 cells to untreated and TNF*α*‐stimulated endothelial cells 24 h post SynC labeling of THP‐1 cells. (A) Representative images shown. (B) Quantification of adherent cells per FOV (n = 3). No differences in the adhesion of SynC‐ labeled and unlabeled THP‐1 cells to TNF*α*‐stimulated endothelial cells. At each time point, the two groups were compared with (B) one‐way ANOVA followed by Tukey's multiple comparison test. * = *p* < 0.05. Data shown as mean ± SD. **Supporting Fig. S2:** Color MPI of 5·10^6^ SynC‐labeled THP‐1 cells either untreated (control) or H_2_O_2_ treated. (A–C) Reconstructed MPI images of side‐by‐side control (left) and H2O2‐treated samples (right) upon applying (A) viable system matrix ($\bar{S_{v}}$) and (B) oxidatively damaged system matrix ($\bar{S_{d}}$). (C) Cell number distributions obtained with $\bar{S_{v}}$ (black) and $\bar{S_{d}}$ (pink), demonstrating a clear differentiation between viable and oxidatively damaged cells in mixed populations. (D–F) Third independent experiment shown. **Supporting Fig. S3:** Color MPI of 1·10^6^ SynC‐labeled THP‐1 cells either untreated (control) or H_2_O_2_‐treated. (A–C) Reconstructed MPI images of side‐by‐side control (left) and H2O2‐treated samples (right) upon applying (A) viable system matrix ( $\bar{S_{v}}$) and (B) oxidatively damaged system matrix ($\bar{S_{d}}$). (C) Cell number distributions obtained with $\bar{S_{v}}$ (black) and $\bar{S_{d}}$ (pink), demonstrating a clear differentiation between viable and oxidatively damaged cells in mixed populations. (D–F) Third independent experiment shown. **Supporting Fig. S4:** Number of cells reconstructed with $\bar{S_{v}}$ and $\bar{S_{d}}$ using color MPI. All individual measurements are shown (n = 3), using total or segmented reconstruction methods. (A–C) MPI of two adjacent samples, each containing (A–C) 5·10^6^ or (D–F) 1·10^6^ SynC‐labeled THP‐1 cells. Reconstruction using the total reconstruction method provided more accurate estimation of the expected cell numbers compared to the segmented reconstruction approach.

## Author Contributions

**Amani Remmo**, **Lena Kampen**, **Antje Ludwig**, **Norbert Löwa**, **Frank Wiekhorst**: study conception and design. **Amani Remmo**, **Lena Kampen**, **Antje Ludwig**, **Norbert Löwa**, **Frank Wiekhorst**, **Olaf Kosch**, **Nike D. C. Fiebig**: methodology. **Lena Kampen**, **Amani Remmo**, **Olaf Kosch**, **Anke Stach**, **Nike D. C. Fiebig**, **Antje Ludwig**: data collection. **Lena Kampen**, **Amani Remmo**, **Olaf Kosch**: formal analysis. **Lena Kampen**, **Amani Remmo**, **Antje Ludwig**, **Frank Wiekhorst**, **Olaf Kosch**: interpretation of results. **Lena Kampen**: writing – original draft preparation. **Lena Kampen**, **Antje Ludwig**, **Amani Remmo**, **Frank Wiekhorst**, **Olaf Kosch**, **Nike D. C. Fiebig**, **Norbert Löwa**: writing – review and editing. **Lena Kampen**, **Olaf Kosch**: visualization. **Amani Remmo**, **Antje Ludwig**, **Frank Wiekhorst**, **Norbert Löwa**: supervision. **Antje Ludwig**, **Frank Wiekhorst**, **Norbert Löwa**: funding acquisition. All authors have read and agreed to the published version of the manuscript.

## Funding

This study was supported by Deutsche Forschungsgemeinschaft (Collaborative research center “Matrix in Vision” (SFB 1340/2) project number: 372486779; Research grant “CellMPI” (project number: 455706279); Deutsches Zentrum für Herz‐Kreislaufforschung (Doctoral scholarship).

## Conflicts of Interest

The authors declare no conflicts of interest.

## Supporting information

Supplementary Material

## Data Availability

The data that support the findings of this study are available from the corresponding author upon reasonable request.
