# Supplementary material for: Magnetic Particle Imaging Distinguishes Viable and Damaged Cells by Exploiting Distinct Magnetic Signatures of Internalized Nanoparticles
Source: Small Sci. 2026 Apr 7;6(4):e202500601. doi: 10.1002/smsc.202500601 (PMC13063793; doi:10.1002/smsc.202500601)
Supplement: Supplementary file 1 — Supplementary Material [file SMSC-6-e202500601-s001.pdf]

Supporting Information

Magnetic Particle Imaging Distinguishes Viable and Damaged Cells by Exploiting Distinct Magnetic Signatures of Internalized Nanoparticles

Lena Kampen [1,2,3*], Olaf Kosch [4], Anke Stach [1,2], Nike D. C. Fiebig [1,2,3], Norbert Löwa[4], Frank Wiekhorst [4], Antje Ludwig [1,2,3*], Amani Remmo [4]

[1] Deutsches Herzzentrum der Charité, Department of Cardiology, Angiology and Intensive Care Medicine, Charitéplatz 1, 10117 Berlin, Germany.

[2] Charité – Universitätsmedizin Berlin, Corporate member of Freie Universität Berlin and Humboldt-Universität zu Berlin, Department of Cardiology, Angiology and Intensive Care Medicine, Charitéplatz 1, 10117 Berlin, Germany.

[3] DZHK (German Centre for Cardiovascular Research), partner site Berlin, Germany.

[4] Physikalisch-Technische Bundesanstalt, Working Group 8.23 Metrology for Magnetic Nanoparticles, Abbestraße 2-12, 10587 Berlin, Germany.

*Correspondence: lena.kampen@dhzc-charite.de; antje.ludwig@dhzc-charite.de

Keywords:

cell physiology imaging, functional cell tracking, immune cell tracking, magnetic particle imaging, magnetic particle spectroscopy

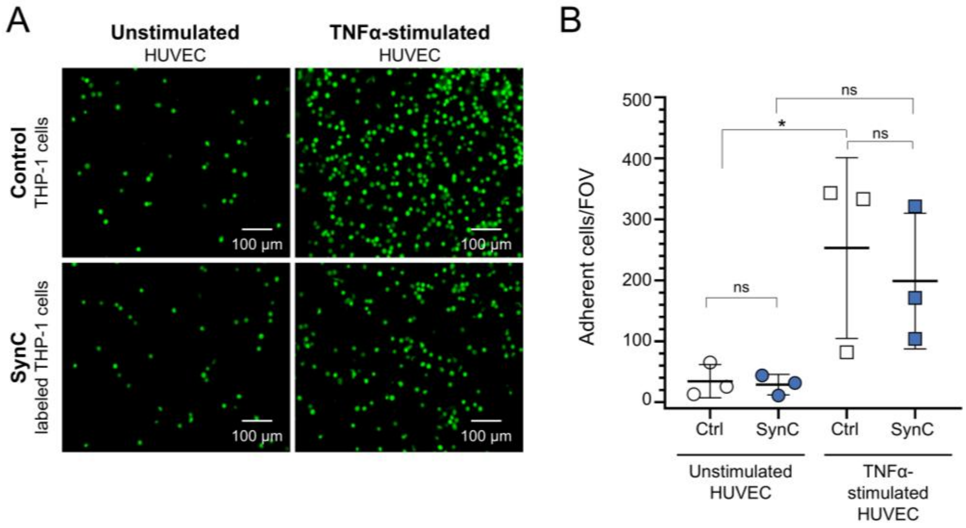

Figure S1: Adhesion of THP-1 cells to untreated and TNFα-stimulated endothelial cells 24 h post SynC labeling of THP-1 cells. (A) Representative images of fluorescent THP-1 cell adhesion shown (20x magnification). (B) Quantification of adherent cells per FOV (n = 3). No differences in the adhesion of SynC- labeled and unlabeled THP-1 cells to TNFα-stimulated endothelial cells. At each time point the two groups were compared with (B) one-way ANOVA followed by Tukey’s multiple comparison test. * = $p < 0.05$. Data shown as mean ± SD. TNFα = Tumor necrosis factor alpha; ANOVA = Analysis of variance; FOV = field of view; SD = standard deviation.

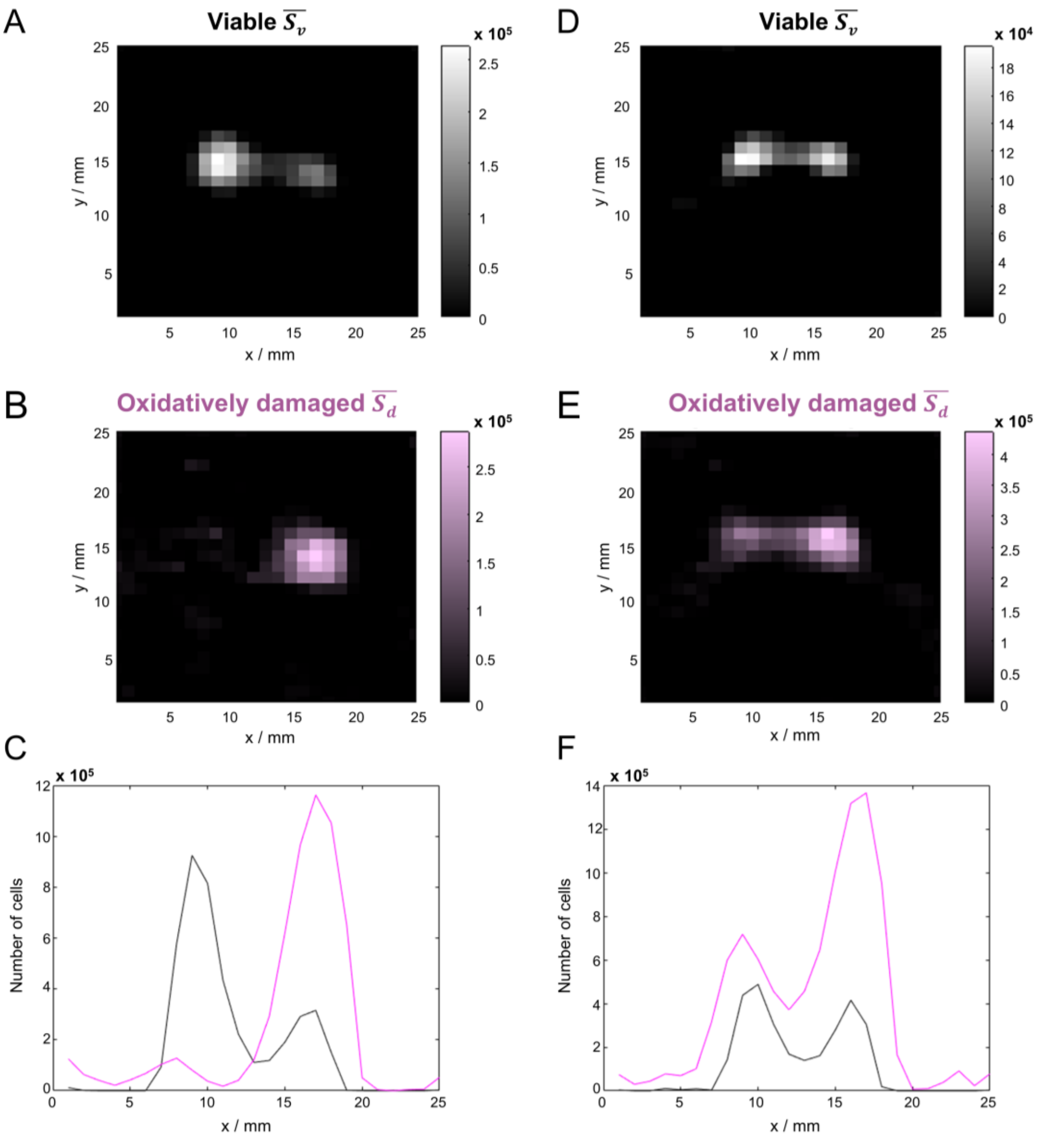

Figure S2: Color MPI of 5·10[6] SynC-labeled THP-1 cells either untreated (control) or H_2O_2-treated. (A–C) Reconstructed MPI images of side-by-side control (left) and H_2O_2-treated samples (right) upon applying (A) viable system matrix ($\overline{S_v}$) and (B) oxidatively damaged system matrix ($\overline{S_d}$). (C) Cell number distributions obtained with $\overline{S_v}$ (black) and $\overline{S_d}$ (pink), demonstrating a clear differentiation between viable and oxidatively damaged cells in mixed populations. (D–F) Third independent experiment shown. MPI = Magnetic particle imaging.

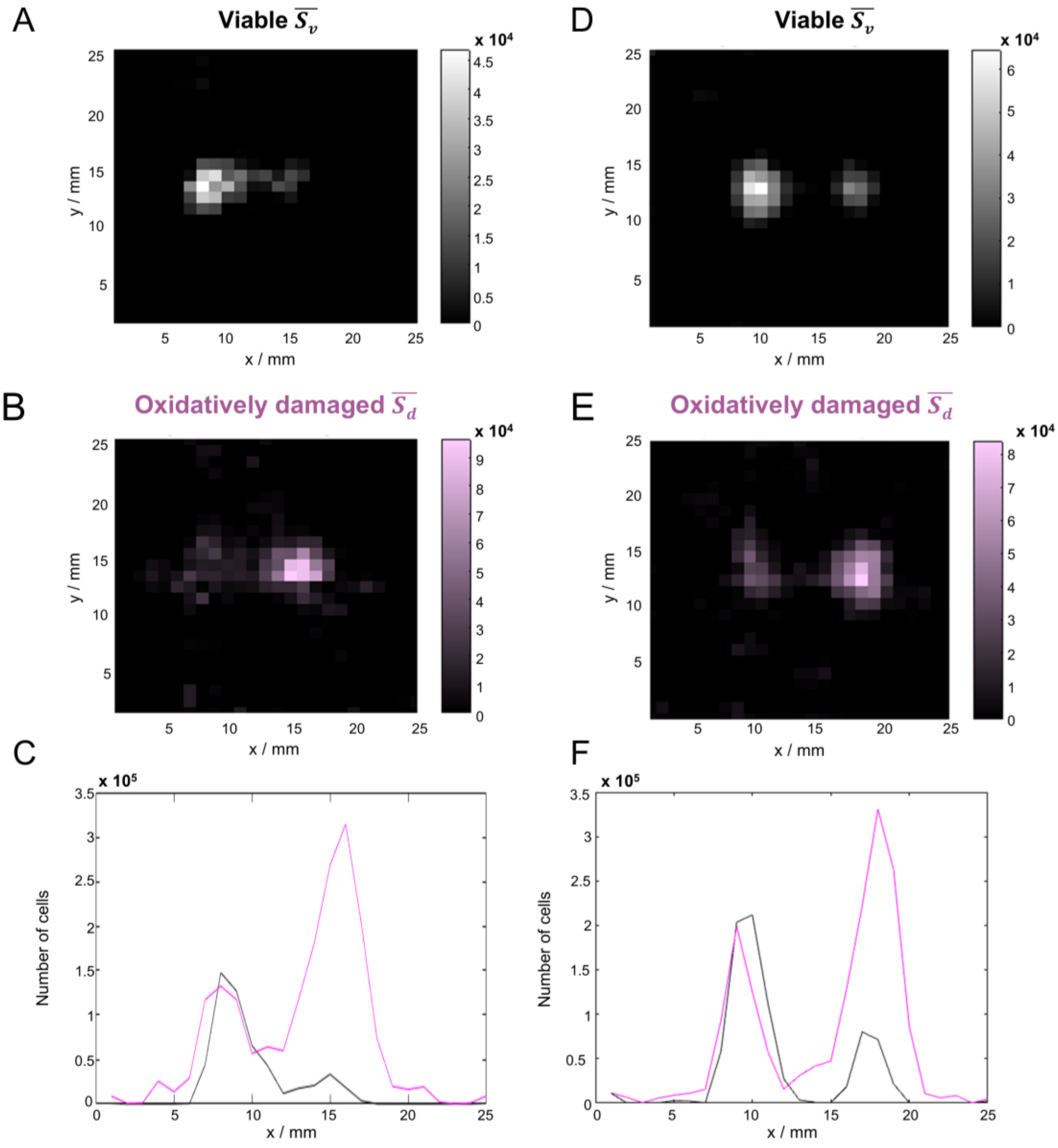

Figure S3: Color MPI of $1 \cdot 10^6$ SynC-labeled THP-1 cells either untreated (control) or H_2O_2-treated. (A-C) Reconstructed MPI images of side-by-side control (left) and H_2O_2-treated samples (right) upon applying (A) viable system matrix ($\overline{S_v}$) and (B) oxidatively damaged system matrix ($\overline{S_d}$). (C) Cell number distributions obtained with $\overline{S_v}$ (black) and $\overline{S_d}$ (pink), demonstrating a clear differentiation between viable and oxidatively damaged cells in mixed populations. (D–F) Third independent experiment shown. MPI = Magnetic particle imaging.

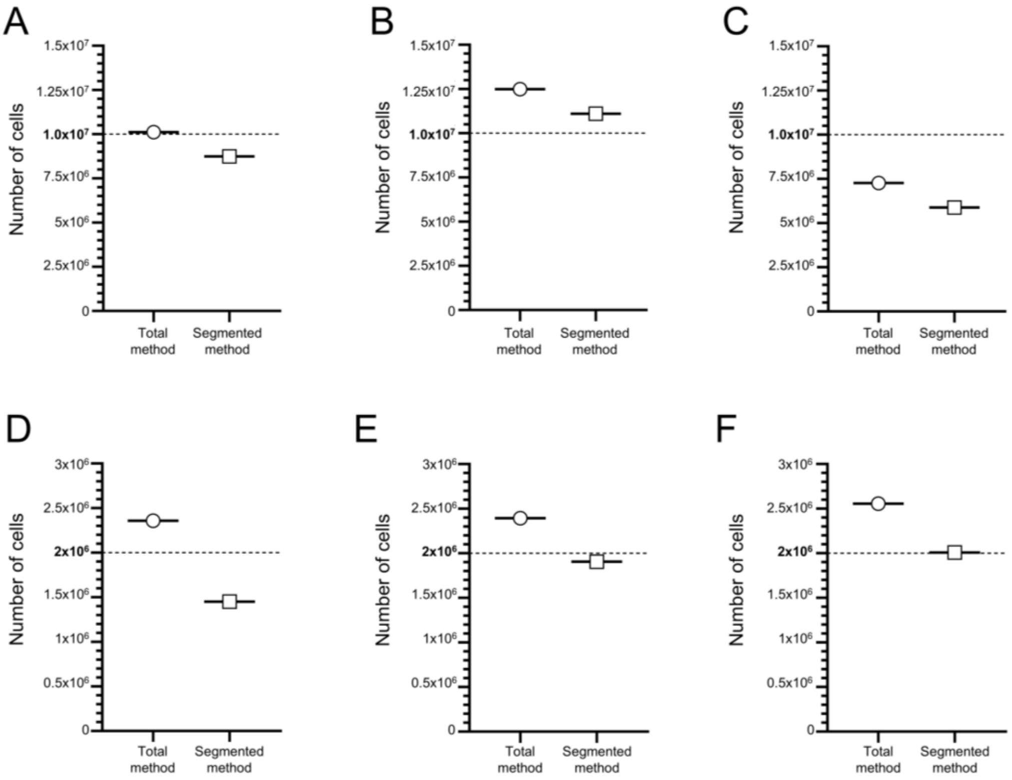

Figure S4: Quantification of cells reconstructed with $\overline{S_v}$ and $\overline{S_d}$ using color MPI. All individual measurements are shown (*n* = 3), using total or segmented reconstruction methods. (A–C) MPI of two adjacent samples, each containing (A–C) $5 \cdot 10^6$ or (D–F) $1 \cdot 10^6$ SynC labeled THP-1 cells. Reconstruction using the total reconstruction method provided more accurate estimation of the expected cell numbers compared to the segmented reconstruction approach. MPI = Magnetic particle imaging.
